# Complex reproductive secretions occur in all extant gymnosperm lineages: a proteomic survey of gymnosperm pollination drops

**DOI:** 10.1007/s00497-018-0348-z

**Published:** 2018-11-14

**Authors:** Natalie Prior, Stefan A. Little, Ian Boyes, Patrick Griffith, Chad Husby, Cary Pirone-Davies, Dennis W. Stevenson, P. Barry Tomlinson, Patrick von Aderkas

**Affiliations:** 1 Centre for Forest Biology, Department of Biology, University of Victoria, Victoria, Canada; 2 Department of Environmental and Plant Biology, Ohio University, Athens, OH USA; 3 Montgomery Botanical Center, 11901 Old Cutler Road, Coral Gables, FL USA; 4 The Arnold Arboretum of Harvard University, 125 Arborway, Boston, MA USA; 5 New York Botanical Garden, Bronx, New York, NY USA

**Keywords:** Gymnosperms, Pollination drops, Cycads, *Ginkgo*, Gnetales, Spermatophytes

## Abstract

*****Key message***:**

**Complex protein-containing reproductive secretions are a conserved trait amongst all extant gymnosperms; the pollination drops of most groups include carbohydrate-modifying enzymes and defence proteins.**

**Abstract:**

Pollination drops are aqueous secretions that receive pollen and transport it to the ovule interior in gymnosperms (Coniferales, Cycadales, Ginkgoales, Gnetales). Proteins are well established as components of pollination drops in conifers (Coniferales) and *Ephedra* spp. (Gnetales), but it is unknown whether proteins are also present in the pollination drops of cycads (Cycadales), *Ginkgo* (Ginkgoales), *Gnetum* (Gnetales), or in the pollination drops produced by sterile ovules occurring on pollen plants in the Gnetales. We used liquid chromatography–tandem mass spectrometry followed by database-derived protein identification to conduct proteomic surveys of pollination drops collected from: *Ceratozamia hildae*, *Zamia furfuracea* and *Cycas rumphii* (Cycadales); *Ginkgo biloba* (Ginkgoales); *Gnetum gnemon* and *Welwitschia mirabilis*, including pollination drops from both microsporangiate and ovulate plants (Gnetales). We identified proteins in all samples: *C. hildae* (61), *Z. furfuracea* (40), *C. rumphii* (9), *G. biloba* (57), *G. gnemon* ovulate (17) and sterile ovules from microsporangiate plants (25) and *W. mirabilis* fertile ovules (1) and sterile ovules from microsporangiate plants (138). Proteins involved in defence and carbohydrate modification occurred in the drops of most groups, indicating conserved functions for proteins in pollination drops. Our study demonstrates that all extant gymnosperm groups produce complex reproductive secretions containing proteins, an ancient trait that likely contributed to the evolutionary success of seed plants.

**Electronic supplementary material:**

The online version of this article (10.1007/s00497-018-0348-z) contains supplementary material, which is available to authorized users.

## Introduction

**Fig. 1:**
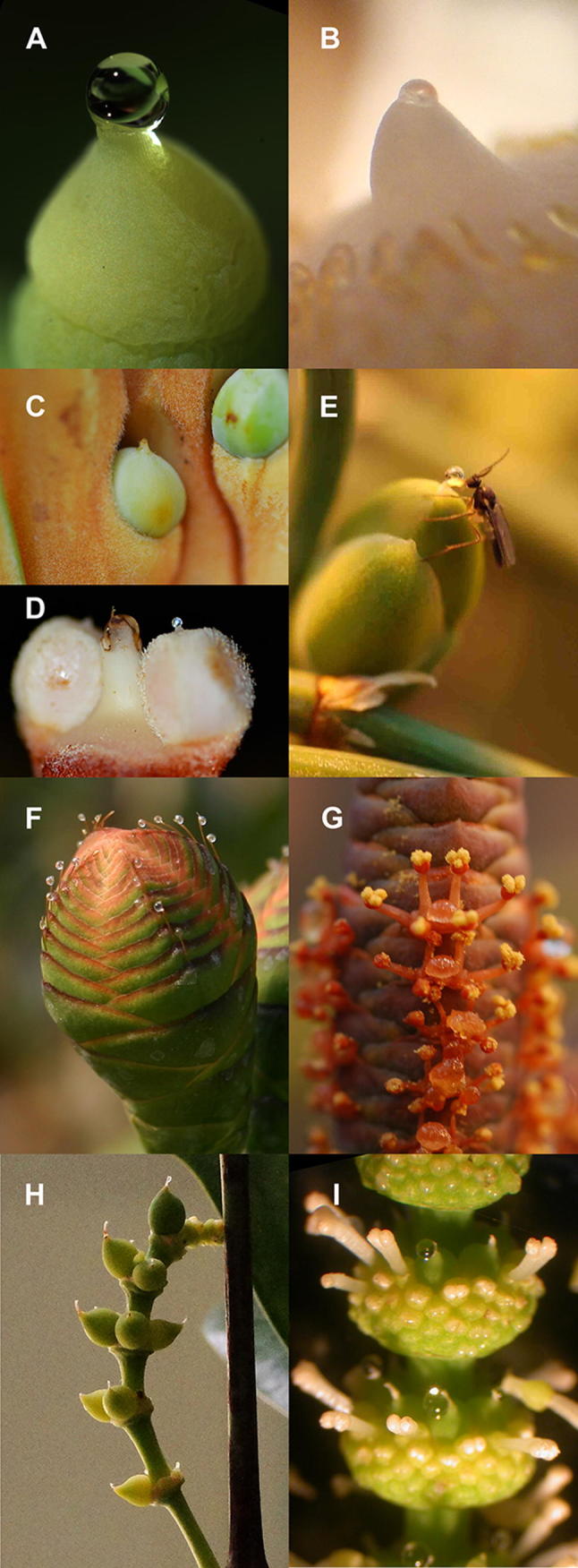
Pollination drops: *Ginkgo biloba* (**a**); *Zamia furfuracea* (**b**); *Cycas rumphii* (**c**); *Ceratozamia hildae* (**d**); *Ephedra monosperma* (**e**); *Welwitschia mirabilis*, female (**f**); *Welwitschia mirabilis*, male (**g**); *Gnetum gnemon*, female (**h**); *Gnetum gnemon*, male (**i**)

Pollination drops are secreted from the ovules of most gymnosperms near the time of pollination (Fig. 1). These minute drops of liquid, 10–1000 nL, play a critical role in gymnosperm reproduction (Prior et al. 2013). Pollination drops receive pollen and move it to the ovule interior where it germinates (Gelbart and von Aderkas 2002). The delivery of pollen directly to the ovule is a characteristic feature of gymnosperms, seed plants whose ovules are exposed around the time of pollination (Tomlinson 2012; Tomlinson and Takaso 2002).

The term gymnosperm is traditionally applied to four groups of extant seed plants, i.e. cycads, *Ginkgo*, Gnetales and conifers, and to many other extinct lineages. Pollination drops are thought to have appeared early in the evolutionary history of seed plants (Doyle 1945; Little et al. 2014; Tomlinson 2012). Cycads (Singh 1978), *Ginkgo* (del Tredici 2007), Gnetales (Carafa et al. 1992; Kato et al. 1995; Singh 1978), as well as all Cupressaceae and Taxaceae *s.l.* share a simple pollination mechanism. When the ovule is receptive for pollination, a pollination drop is secreted and exudes through the micropyle. Pollen is delivered to the ovule by wind, or by insects in the case of some cycads (Terry et al. 2005) and Gnetales (Kato 1994), and possibly in *Ginkgo* (Nepi et al. 2017). Pollination drop and pollen then meet, and pollen is carried through the micropyle as the pollination drop recedes. The pollen comes to rest on the nucellus where it germinates. Although most conifers share this simple mechanism (Gelbart and von Aderkas 2002), other conifers have complex mechanisms that involve unique adaptations of morphology and physiology (Leslie 2010; Owens et al. 1998; von Aderkas and Leary 1999). A few conifers, e.g. all Araucariaceae (Haines et al. 1984) and some Pinaceae and Podocarpaceae (Doyle 1945), do not produce pollination drops at all. Pollen germinates *ex ovulo*. The growing pollen tubes find the ovules. Some Gnetales, e.g. *Gnetum gnemon* (Carmichael and Friedman 1996), *Welwitschia mirabilis* (Wetschnig and Depisch 1999) and *Ephedra fragilis* (Celedón-Neghme et al. 2016), secrete pollination drops from sterile ovules that develop amongst the microsporangia in pollen strobili in addition to the drops produced by exclusively ovulate plants. These pollination drops also appear around the time of microsporangia dehiscence of the pollen strobili.

Biochemical analyses of pollination drops have revealed the presence of minerals and organic molecules within these aqueous secretions (Gelbart and von Aderkas 2002). Calcium (Fujii 1903; von Aderkas et al. 2012), phosphates (Ziegler 1959), sugars and amino acids (Nepi et al. 2017) have been identified in an array of gymnosperm taxa. More recently, proteins have been discovered in seven conifer species (O’Leary et al. 2007; Pirone-Davies et al. 2016; Poulis et al. 2005; Wagner et al. 2007), in seven species of *Ephedra* (von Aderkas et al. 2014) and in *Welwitschia mirabilis* (Wagner et al. 2007). Database-derived identifications of these proteins in conifers, *Ephedra* and *W. mirabilis*, indicate that they are secreted proteins mainly involved in carbohydrate modification (e.g. invertase, glucanase) or in defence (e.g. chitinase, peroxidase) (Pirone-Davies et al. 2016; O’Leary et al. 2007; Poulis et al. 2005; von Aderkas et al. 2014; Wagner et al. 2007). In *Ephedra* (von Aderkas et al. 2014) and *Cephalotaxus* (Pirone-Davies et al. 2016), cytoplasmic proteins attributed to the degradation of the nucellus during pollen chamber formation were detected in addition to secreted proteins.

The biochemical complexity of pollination drops implies that their functions extend beyond pollen capture, transport and hydration. It is likely that calcium, sugars and amino acids serve as nutrients during pollen germination and microgametophyte development in all species and that sugars and amino acids serve as a reward in insect-pollinated species (Little et al. 2014). Experiments with pollination drop proteins suggest that pollination drops could house a more dynamic biochemical network than previously considered. Two enzymatic functions have been demonstrated in *Pseudotsuga* pollination drops so far. An invertase is thought to exert control over pollen germination by regulating the sugar composition of the pollination drop, favouring congeneric pollen (von Aderkas et al. 2012), and a chitinase is thought to control microbial contamination by cleaving chitin, a common component of fungal cell walls (Coulter et al. 2012).

Our current knowledge of pollination drop proteins is incomplete. It is unknown whether pollination drops of cycads, *Ginkgo* or *Gnetum* (Gnetales) contain proteins, or whether those secreted from sterile ovules on microsporangiate strobili of Gnetales contain proteins. Proteomic surveys of pollination drops from these gymnosperm lineages are needed to begin to understand the role of protein biochemistry during the first contact between pollen and ovule, and to better evaluate the significance of pollination drop biochemistry during the evolution of seed plant reproduction.

Increased availability of sequence information for non-model plants combined with extremely sensitive mass spectrometry instruments and improved database search algorithms for cross-species protein identification has made deep proteomic surveys in gymnosperms possible for the first time. Here, we used liquid chromatography–tandem mass spectrometry (LC–MS/MS) along with a custom gymnosperm protein database derived from transcriptomic data to conduct proteomic surveys in pollinations drops of cycads (*Ceratozamia hildae*, *Zamia furfuracea*, *Cycas rumphii*), Ginkgo (*Ginkgo biloba*) and Gnetales (*Gnetum gnemon*, *Welwitschia mirabilis*). Our analyses of *Gnetum* and *Welwitschia* included proteomic surveys of pollination drops collected from both fertile ovules from ovulate plants, and sterile ovules from microsporangiate plants.

## Methods

### Pollination drop collection

Pollination drops were collected directly from the ovules of *Cycas rumphii*, *Ginkgo biloba*, *Gnetum gnemon* (fertile and sterile ovules) and *Welwitschia mirabilis* (fertile and sterile ovules). The nanolitre volumes of individual pollination drops necessitated pooling samples from single or multiple plants (Online Resource 1) to achieve the minimum threshold volumes needed for further processing for mass spectrometric analysis. For *Ceratozamia hildae* and *Zamia furfuracea*, megasporophylls were removed from the central cone axis and placed into a humid chamber prior to drop collection. For all taxa, pollination drops were collected using either 10-μL micropipette tips or flame-pulled 10-μL glass capillary tubes. Samples were expelled into 1.5-mL micro-centrifuge tubes or 1.5-mL Eppendorf tubes. Great care was taken to avoid keratin contamination during collection. Drops were stored at − 20 °C until processing for mass spectrometry analysis.

### Proteomics

#### Creation of the Gymno_DB database

Gymnosperm RNA-Seq data were collected from the following resources: published transcriptome assembly from the Natural Sciences and Engineering Research Council of Canada Strategic Grant *Megastigmus* and Conifers: The Biology of Invasion (one species; Little et al. 2016); unpublished, unassembled reads from the New York Plant Genomics Consortium United States National Science Foundation grant IOS-0922738 (three species); publicly available transcriptomes from the Dendrome Project (nine species); unassembled reads from the 1KP project (84 species; Johnson et al. 2012; Matasci et al. 2014; Wickett et al. 2014; Xie et al. 2014). Information pertaining to the species, tissue types and sources is provided in Online Resource 2. A total of 97 RNA-Seq data sets representing 91 species (six species with two transcriptome assemblies) were included. Representatives from all major extant gymnosperm clades were included.

In the case of species for which there were only unassembled RNA-seq reads, we produced a transcriptome assembly following Little et al. (2016), which is included in this study. The resulting 97 transcriptome assembly FASTA files were modified to produce unique headers across the study and then combined into a single FASTA file. Then, we used the program EMBOSS tool GetORF (MRC Rosalind Franklin Centre for Genomics Research, Wellcome Trust Genome Campus, Cambridge, UK) to predict all possible open reading frames and then translate these possible open reading frames into amino acid sequences. The following qualifiers were used: Table 1 (Standard with alternative Initiation codons); Find 1 (Translation of regions between START and STOP codons).

**Table 1:** Summary of proteins found in the pollination drops from ovules borne on ovule-only fertile structures of *Ceratozamia hildae* (CH), *Zamia furfuracea* (ZF), *Cycas rumphii* (CR), *Ginkgo biloba* (GB), *Gnetum gnemon* (GG) and *Welwitschia mirabilis* (WM)

Type	Protein name	Number of proteins					
		CH	ZF	CR	GB	GG	WM
Carbohydrate modifying	Glycosyl hydrolase	8	4	–	3	1	–
	Alpha-galactosidase	1	2	–	1	2	–
	Beta-xylosidase	2	2	–	1	–	–
	Beta-glucosidase	2	–	–	1	–	–
	Cellulase	1	–	–	1	–	–
	Beta-hexosaminidase	–	1	–	1	–	–
	Beta-galactosidase	4	–	–	–	–	–
	Pectin lyase-like protein	3	–	–	–	–	–
	Alpha-xylosidase	2	–	–	–	–	–
	Alpha-fucosidase	–	–	–	–	1	–
	Beta-glucanase	–	–	–	–	1	–
Defence response	Chitinase	1	11	1	–	1	–
	Osmotin/thaumatin	1	5	–	3	1	–
	HOPZ-activated resistance	–	–	–	–	–	1
Lipid transfer	Lipid transfer protein	1	–	2	1	1	–
Plant-type cell wall organization	Expansin	–	–	–	1	–	–
Oxidation–reduction	FAD-binding Berberine family protein	2	1	–	–	3	–
	Peroxidase	2	–	2	–	1	–
	SKU5 similar	4	–	–	–	–	–
	Laccase	1	–	–	–	–	–
	d-Arabinono-1,4-lactone oxidase family protein	–	3	–	–	–	–
Proteolysis	Aspartyl protease	1	2	–	6	1	–
	Cysteine proteinase	4	1	–	1	–	–
	Subtilisin-like serine endopeptidase	3	5	–	–	1	–
	Serine carboxypeptidase	3	–	–	–	–	–
	Alpha/beta-hydrolases	2	–	–	–	–	–
	Aleurain-like protease	1	–	–	–	–	–
Translation	Elongation factor	–	1	–	8	–	–
	RAB GTPase	–	–	–	1	–	–
	Ribosomal protein	–	–	–	2	–	–
ATP metabolic process	ATP synthase	–	–	–	2	–	–
Glycolysis	Enolase	–	–	–	2	–	–
	Aldolase	–	–	–	1	–	–
	Phosphoglycerate kinase	–	–	–	1	–	–
	Pyruvate kinase	–	–	–	1	–	–
Protein phosphorylation	Leucine-rich repeat transmembrane protein kinase	1	1	–	–	–	–
	Receptor-like protein kinase	1	–	–	–	–	–
Various extracellular	Fasciclin-like arabinogalactan protein	1	–	–	1	–	–
	GAST1 protein homolog	1	–	–	–	–	–
	Glucuronidase	1	–	–	–	–	–
	Phosphatase	1	–	–	–	–	–
	SIT4 phosphatase-associated family protein	–	–	1	–	–	–
	Alpha-amylase	–	–	–	2	–	–
	Glutathione *S*-transferase family protein	–	–	–	1	–	–
	Cystatin B	–	–	–	–	1	–
	EGG CELL 1.2 protein	–	–	–	–	1	–
Various intracellular	SHV3-like protein	1	1	–	–	–	–
	Ubiquitin	–	–	1	1	–	–
	Immunoglobulin E-set superfamily protein	1	–	–	–	–	–
	P-loop containing nucleoside triphosphate hydrolases superfamily protein	1	–	–	–	–	–
	Phosphoenolpyruvate carboxylase	1	–	–	–	–	–
	Serine transhydroxymethyltransferase	1	–	–	–	–	–
	Uclacyanin	1	–	–	–	–	–
	Calcium/calmodulin-dependent serine/threonine-kinase	–	–	1	–	–	–
	SIT4 phosphatase-associated family protein	–	–	1	–	–	–
	CAP protein	–	–	–	2	–	–
	RNA-binding protein	–	–	–	2	–	–
	Calreticulin	–	–	–	1	–	–
	Development and cell death domain protein	–	–	–	1	–	–
	Embryonic cell protein	–	–	–	1	–	–
	Glyoxal oxidase-related protein	–	–	–	1	–	–
	Glutamine amidotransferase	–	–	–	1	–	–
	Heat shock protein	–	–	–	1	–	–
	Late embryogenesis abundant protein family protein	–	–	–	1	–	–
	Phospholipase	–	–	–	1	–	–
	RAN GTPase 3	–	–	–	1	–	–
	S-adenosylmethionine synthetase family protein	–	–	–	1	–	–
Unknown	AT5G23200.1	–	–	–	–	1	–
Total proteins		61	40	9	57	17	1

All of the resulting open reading frames were concatenated into one FASTA file along with the Common Repository of Adventitious Proteins database version 2012.01.01 (cRAP, The Global Proteome Machine Organization). The final database contained 62 148 544 predicted amino acid sequences, each with a unique header that includes the source species, and original RNA transcript ID. This database, hereafter referred to as Gymno_DB, was verified by PEAKS 6 and uploaded to the PEAKS Online 6 server located at the University of Victoria Genome British Columbia Proteomics Centre (Victoria, Canada).

#### Mass spectrometry analysis

Pollination drop samples were reduced with dithiothreitol for 30 min at 37 °C and alkylated with iodoacetamide at 37 °C in darkness for 30 min. Samples were then digested with 2 μg trypsin (Promega, Madison, WI, USA) at 37 °C for 16 h. A Waters HLB Oasis column (Milford, MA, USA) was used to desalt the samples. The samples were concentrated using a Speed Vac (Thermo Fisher Scientific, Bremen, Germany) and then stored at − 80 °C until analysis.

For liquid chromatography–tandem mass spectrometry analysis (LC–MS/MS), a 100 μL sample of the peptide mixture was rehydrated with 2% acetonitrile/water/2% formic acid. Samples were separated by online reversed-phase chromatography using a Thermo Scientific EASY-nLC II system (Thermo Fisher Scientific) with an in-house prepared reversed-phase pre-column Magic C-18AQ (100 μm in diameter, 2 cm length, 5 μm, 100 Å, Michrom BioResources Inc., Auburn, CA) and an in-house prepared reversed-phase nano-analytical column Magic C-18AQ (75 μm in diameter, 15 cm length, 5 μm, 100 Å, Michrom BioResources Inc.). The flow rate was 300 nl/min. An LTQ Orbitrap Velos mass spectrometer with a Nanospray II source (Thermo Fisher Scientific) was coupled to the chromatography system. Two solvents were used: solvent A consisted of 2% acetonitrile and 0.1% formic acid; solvent B consisted of 90% acetonitrile and 0.1% formic acid. A 249 bar (~ 5 μL) pre-column equilibration and a 249 bar (~ 8 μL) nano-column equilibration were performed. The samples were then separated by a 90-min gradient (0 min: 5% solvent B; 80 min: 45% solvent B; 2 min: 90% solvent B; 8 min: 90% solvent B). The following parameters were used on the LTQ Orbitrap Velos (Thermo Fisher Scientific): nano-electrospray ion source with spray voltage 2.2 kV; capillary temperature 225 °C; survey MS1 scan *m*/*z* range 400–2000 profile mode; resolution 60,000 @ 400 *m*/*z* with AGC target 1E6; one microscan with maximum inject time 200 ms; Lock mass Siloxane 445.120024 for internal calibration with preview mode for FTMS scans: ON; injection waveforms: ON; monoisotopic precursor selection: ON; rejection of charge state 1. The five most intense ions (charge state 2–4) exceeding 5000 counts were selected for a 7500-resolution high collision dissociation (HCD) (FT MSMS fragmentation scans 2–6). Detection was in profile mode. Dynamic exclusion settings were: repeat count: 2; repeat duration: 15 s; exclusion list size: 500; exclusion duration: 60 s with a 10-ppm mass window. The HCD activation isolation window was: 2 Da; AGC target: 1E5; maximum inject time: 500 ms; activation time: 0.1 ms; activation Q: 0.250 HCD collision energy: 30%.

#### Data analysis parameters

Raw LC–MS/MS data were converted into Mascot generic format (MGF) using Proteome Discoverer 1.4 (Thermo Fisher Scientific). MGF files were searched using PEAKS 6 (Bioinformatics Software Inc.) against our in-house database, Gymno_DB. PEAKS DB incorporates traditional database searching with de novo sequencing, and the homology search tool SPIDER. PEAKS DB + SPIDER searches were performed using the following parameters: parent mass error tolerance: 8.0 ppm; fragment mass error tolerance: 0.03 Da; precursor mass search type: monoisotopic; enzyme: trypsin; max-missed cleavages: 1; non-specific cleavage: none; fixed modifications: carbamidomethylation 57.02; variable modifications: deamidation (NQ) 0.98, oxidation (M) 15.99; max variable PTM per peptide: 3.

PEAKS 6 software includes a results validation program. It uses a decoy fusion method to calculate the false discovery rates (FDRs) at the peptide spectrum match (PSM), peptide and protein levels (Zhang et al. 2012). All results were filtered with an FDR of ≤ 1% at the PSM, peptide and protein levels, and a requirement for at least one unique peptide per protein identification.

#### Protein identification

BLASTP (BLAST 2.2.28+, National Centre for Biotechnology Information) was used to annotate the predicted open reading frames (proteins) identified as matches to pollination drop mass spectrometry data. PEAKS 6 was used to generate FASTA files containing the amino acid sequences corresponding to the full-length open reading frames of all hits for each LC–MS/MS run. Each FASTA file was blasted against the *Arabidopsis* Information Resource (TAIR 10) protein database accessed online from ftp://ftp.arabidopsis.org/home/tair/Proteins/TAIR10_protein_lists/. BLASTP was run with standard parameters using the best-hit algorithm. These BLASTP searches used computing resources provided by WestGrid and Compute/Calcul Canada. BLASTP hits were filtered at a threshold *E*-value < e−5. Descriptive names and gene ontology (GO) annotations for the TAIR10 gene models (Berardini et al. 2004) were collected from the file ATH_GO_GOSLIM.txt.gz (03/09/2013) accessible on the website ftp://ftp.arabidopsis.org/home/tair/Ontologies/Gene_Ontology/.

## Results and discussion

Proteomic analyses of pollination drops from cycads, *Ginkgo* and female cones of the Gnetales identified: 61 proteins in *C. hildae*, 40 proteins in *Z. furfuracea* and 9 proteins in *C. rumphii*; 57 proteins in *G. biloba*; 17 proteins in *G. gnemon*; 1 protein in *W. mirabilis* (Table 1). Analyses of pollination drops of male gnetalean cones identified: 138 proteins in *W. mirabilis* (Table 2) and 25 proteins in *G. gnemon* (Table 3). Protein accessions, PEAKS scores, TAIR gene models based on BLASTp matches, and their *E*-values, as well as the corresponding gene ontology (GO) annotations for biological process and cellular component are given for all species in Online Resource 3. Several detected proteins did not have a BLASTp hit, or had a BLASTp > 1e−25. Our discussion here will focus on annotations based on BLASTp ≤ 1e−25.

**Table 2:** Summary of proteins found in the male pollination drops of sterile ovules in pollen cones of *Welwitschia mirabilis*

Protein name	Number of proteins
Ribosomal proteins	21
Dehydrogenases	12
Heat shock proteins	10
Chaperonins/chaperone proteins	9
Kinases	8
Peroxidases	7
ATP synthase proteins/ATPases	5
Isomerases	5
Mitochondrial proteins	4
Synthetases/ligases	4
GTP-binding elongation factors	3
Reductases	3
Acyl-CoA-binding proteins	2
Calreticulins	2
Clathrin-related proteins	2
Coatomers	2
Serine hydroxymethyltransferases	2
Synthase	2
Tubulin proteins	2
UDP-glucose pyrophosphorylases	2
Actin depolymerizing factor	1
Actin protein	1
Acyl carrier protein	1
ADP/ATP carrier	1
Annexin	1
Aspartate aminotransferase	1
Cold, circadian rhythm and RNA-binding protein	1
DEA(D/H)-box RNA helicase	1
Enolase	1
Eukaryotic translation initiation factor	1
General regulatory factor	1
Importin	1
KH domain-containing protein/zinc finger family protein	1
MD-2-related lipid recognition domain-containing protein	1
Metallopeptidase	1
NmrA-like negative transcriptional regulator family protein	1
Nucleotide-diphospho-sugar transferase	1
Peroxisomal 3-ketoacyl-CoA thiolase	1
Phosphoglucomutase/phosphomannomutase	1
Phosphoglycerate mutase, 2,3-bisphosphoglycerate-independent	1
Profilin	1
RNA-binding plectin/S10 domain-containing protein	1
Secretion-associated RAS superfamily protein	1
Single hybrid motif superfamily protein	1
Superoxide dismutase	1
Thioredoxin	1
Transketolase family protein	1
Translationally controlled tumour protein	1
TUDOR-SN protein	1
Ubiquitin-fold modifier	1
Xyloglucan endotransglucosylase/hydrolase	1
Total	140

**Table 3:** Summary of proteins found in male pollination drops of ovules in pollen bearing cones of *Gnetum gnemon*

Protein name	Number of proteins
GTP-binding elongation factor Tu family protein	3
ATPase subunit	3
FAD-binding Berberine family protein	2
Cysteine proteinase	1
DNA mismatch repair protein MutS	1
ER-type Ca^2+^-pumping ATPase	1
Fumarase	1
Glutamate dehydrogenase	1
Homolog of yeast autophagy 18	1
Hydroxyproline-rich glycoprotein family protein	1
Immunoglobulin E-set superfamily protein	1
Isoflavone reductase	1
Lumenal cyclophilin	1
Plastidic alpha-glucan phosphorylase	1
Poly(ADP-ribose) polymerase	1
Ribosomal S11 family protein	1
Serine carboxypeptidase-like protein	1
Subtilisin-like serine protease	1
Translation protein SH3-like family protein	1
Translation elongation factor 2-like protein	1
Total	25

### Cycads

Our results demonstrate, for the first time, that proteomes exist within cycad pollination drops. Analyses identified many proteins in *Ceratozamia*, *Zamia* and *Cycas*, genera that represent two of the three extant families in this clade (The Gymnosperm Database 2018). Analysis revealed that both extracellular proteins (protein secretome) and intracellular, cytoplasmic proteins (protein degradome) are present. GO annotations revealed that most are extracellular proteins, forming protein secretomes like those previously described in conifers (O’Leary et al. 2007; Pirone-Davies et al. 2016; Poulis et al. 2005; Wagner et al. 2007) and *Ephedra* spp. (von Aderkas et al. 2014). The protein classes represented in the secretome include cell wall/carbohydrate-modifying proteins, defence proteins and pollen recognition proteins. Not all proteins found in drops are expressly secreted into drops. Because proteins are washed from degrading nucellar cells into the pollination drops during pollen chamber formation, intracellular proteins were also detected. For example, proteins found compartmentalized in cellular organelles, such as mitochondria, are not secreted into the plant’s apoplast; they only get into the apoplast following cell breakdown. When we found such proteins, they were assigned to the degradome. To date, such protein degradomes have been described in *Ephedra* spp. (von Aderkas et al. 2014) and *Cephalotaxus* spp. (Pirone-Davies et al. 2016) and include proteins such as elongation factor 1-α, histones, calmodulin and glycosyl hydrolases as well as storage proteins. Interestingly, GO annotations also indicated at least one plasma membrane protein present, a leucine-rich repeat transmembrane receptor-like kinase (RLK). These RLK proteins have diverse functions in plant growth, response to stress and detection of exogenous signals (Mizuno et al. 2007). Although RLKs are unlikely to function within pollination drops, this discovery may support recent suggestions generated from behavioural studies of cupressaceous pollination drops (Dörken and Jagel 2014) and from gene expression studies of *Cephalotaxus sinensis* (Pirone-Davies et al. 2016) that interactions occur between drop components and the nucellar surface.

The best-represented group of proteins in cycad pollination drops were those involved in carbohydrate modification processes. Some of these proteins have been identified previously in conifers and *Ephedra* spp., for example alpha- and beta-galactosidases (Pirone-Davies et al. 2016; Poulis et al. 2005; von Aderkas et al. 2014), beta-glucosidases (Pirone-Davies et al. 2016; von Aderkas et al. 2014; Wagner et al. 2007) and xylosidases (Poulis et al. 2005; von Aderkas et al. 2014). These enzymes are often involved in cell wall restructuring and in pollination drops that are thought to act on pollen cell walls to facilitate pollen germination and pollen tube growth (Poulis et al. 2005; Wagner et al. 2007). Two additional cell wall-modifying enzymes were detected in cycad pollination drops that have not been found in other gymnosperms: a pectin lyase-like protein (*C. hildae*) and a beta-hexosaminidase (*Z. furfuracea*). In general, cell wall-modifying enzymes may also contribute to the formation of pollen chambers. Cycads and *Ginkgo* are zooidogamous species, which can be distinguished from siphonogamous species by their flagellated sperm. The formation of a pollen chamber by enzymatic breakdown in cycads and *Ginkgo* allows their pollen tubes to be positioned for sperm release above the archegonia (Loconte and Stevenson 1990).

Pollination drop proteins may play a role in nutrient mobilization for the emerging pollen tube (Poulis et al. 2005; Wagner et al. 2007). Invertases convert sucrose to fructose and glucose for uptake by the pollen tube. Invertases have been shown to be functional in *Pseudotsuga menziesii* drops in vitro (von Aderkas et al. 2012). In our cycad samples, a family 32 glycosyl hydrolase with possible invertase activity was detected in *Z. furfuracea*. Tang (1987, 1993) detected sucrose and glucose in pollination drops of the cycads *Zamia pumila* and *Ceratozamia robusta*. Determining whether these sugars are regulated by invertase would require in vitro assays.

Proteases may also mobilize nutrients within pollination drops by liberating amino acids for uptake by the growing pollen tube (Poulis et al. 2005; Wagner et al. 2007). A number of proteases have been identified previously in conifers (Poulis et al. 2005; Wagner et al. 2007) and some *Ephedra* species (von Aderkas et al. 2014). Several proteases were also detected in *C. hildae* and/or *Z. furfuracea*, including aspartyl protease, subtilisin-like serine endopeptidase, cysteine proteinase, alpha/beta-hydrolase and serine carboxypeptidase.

Defence from pathogens is believed to be a key role for pollination drop proteins given the exposed nature of pollination drops and their nutrient-rich composition (O’Leary et al. 2007; Poulis et al. 2005; Wagner et al. 2007). A ‘cocktail’ of defence proteins has been reported for conifers and gnetaleans including chitinases, thaumatin-like proteins and peroxidases (O’Leary et al. 2007; Pirone-Davies et al. 2016; Poulis et al. 2005; Wagner et al. 2007; von Aderkas et al. 2014). Chitinases work to break down chitin in fungal cell walls (Grover 2012), inhibit bacterial growth (Majeau et al. 1990) and elicit up-regulation of defence pathways (Boller 1995; Grover 2012; Shibuya and Minami 2001). Chitinolytic activity was detected within *Pseudotsuga menziesii* drops through biochemical and in-gel assays (Coulter et al. 2012). Chitinases were also present in *C. hildae*, *Z. furfuracea* and *C. rumphii* possibly suggesting a similar function in cycads. Thaumatin-like proteins also act as anti-fungal agents by disturbing fungal wall formation (Zareie et al. 2002) or inhibiting fungal enzymes themselves (Fierens et al. 2007). Thaumatin-like proteins were detected in *C. hildae* and *Z. furfuracea* perhaps with a similar role. Peroxidases are also known as defence proteins (Kawano 2003) and were detected in both *C. hildae* and *C. rumphii.* Additionally, a number of proteins involved in reduction–oxidation reactions were found: FAD-binding Berberine family protein, d-arabino-1,4-lactone oxidase family protein, laccase 5, SKU5 similar protein. In *Nicotiana* nectar, a network of redox proteins, some similar to those found in cycads, works to create an antimicrobial environment by establishing a high concentration of hydrogen peroxide (Park and Thornburg 2009). Whether a similar network of proteins is at work in cycad pollination drops would require extensive biochemical investigation.

Lipid transfer proteins (LTPs) were detected in both *C. hildae* and *C. rumphii* and previously in *Larix* pollination drops (O’Leary 2004). Lipid transfer proteins have a broad range of functions, from defence against bacterial and fungal pathogens (Maldonado et al. 2002; Nielsen et al. 1996; Regente et al. 2005) to wax, suberin and sporopollenin deposition (Edstam et al. 2013). Interestingly, LTPs are also thought to be involved in pollen tube adhesion to the stigma and style in *Lillium longiflorum* Thunb (Park et al. 2000).

A collection of proteins found in cycad pollination drops fall into the category of arabinogalactan proteins (Seifert and Roberts 2007). These include fasciclin-like arabinogalactan protein 8, uclacyanin 1, SKU5 similar 4 and 5 proteins, and SHV3-like 1 protein with glycerophosphodiesterase activity. In angiosperms, arabinogalactan proteins are found in reproductive tissues and interact with pollen by acting as an adhesive (Clarke et al. 1979), influencing self-recognition (Lind et al. 1994) and defining a path for pollen tube growth (Cheung et al. 1995). Arabinogalactan proteins were localized to the nucellus in *Taxus*, and their concentration peaked synchronously with pollination drop production (O’Leary et al. 2004). O’Leary et al. (2004) hypothesized that since arabinogalactan proteins originate in the *Taxus* nucellus, they may also play a role in pollen tube guidance. Further work is needed to confirm this possibility.

Cycads are a distinct lineage of seed plants with a special set of reproductive characteristics. Unlike conifers and gnetaleans, they are zooidogamous, i.e. produce flagellate sperm, and have haustorial pollen tubes (Chamberlain 1919; Choi and Friedman 1991). In addition, many cycad species are insect-pollinated (Stevenson et al. 1998; Tang 1987). Yet despite their distinctiveness, similarities were observed between cycad pollination drop secretomes and those seen previously in conifers and gnetaleans, namely proteins involved in carbohydrate modification and pathogen defence. This further supports the hypothesis presented by Wagner et al. (2007) of conserved functions for pollination drop proteins.

### *Ginkgo*

Nearly five dozen proteins were identified in this first proteomic survey of *G. biloba* pollination drops. Protein analysis revealed that both a secretome and a degradome are present. *Ginkgo biloba* develops a deep pollen chamber in the nucellus during drop production (Friedman 1987), probably accounting for a degradome with a number of cytoplasmic proteins. There were also a number of proteins with annotations to the plasma membrane.

The pollination drop proteome of *G. biloba* included proteins similar to those found previously in cycads in our study reported here, as well as in conifers (O’Leary et al. 2007; Pirone-Davies et al. 2016; Poulis et al. 2005; Wagner et al. 2007) and *Ephedra* (von Aderkas et al. 2014). These included a complement of carbohydrate-modifying proteins: galactosidase, xylosidase, cellulase, hexosaminidase, glucosidase and glycosyl hydrolase. These enzymes likely influence pollen germination and pollen tube growth through cell wall reorganization. As in *Cephalotaxus* (Pirone-Davies et al. 2016), alpha-amylase was also found in *G. biloba*. In some gymnosperms, starch accumulates in nucellar cells around drop production. Pirone-Davies suggested a potential role for the alpha-amylase in *Cephalotaxus* in liberating bond energy by breaking down starch. As seen in other gymnosperms, *G. biloba* pollination drops also included defence proteins (osmotin/thaumatin-like proteins), proteolytic enzymes (aspartyl protease and cysteine protease) and a fasciclin-like arabinogalactan protein.

A novel protein found in *G. biloba* pollination drops was an alpha-expansin. Expansins are extracellular proteins involved in cell wall expansion (Lipchinsky 2013) and have known roles in reproduction in angiosperms (Valdivia et al. 2009; Zaidi et al. 2012). In *Triticale* (× *Triticosecale* Wittmack), expansins diffuse from pollen and loosen the cell walls of stigma tissue, making it easier for the pollen tube to penetrate (Zaidi et al. 2012). Two plausible roles for expansins in pollination drops could be to facilitate pollen tube cell wall expansion and/or to soften nucellar tissue, thereby facilitating haustorial pollen tube penetration.

A number of proteins involved in translation, glycolysis and other intracellular processes (e.g. RNA-binding KH domain-containing protein, calreticulin 1a, heat shock proteins) were detected in *G. biloba* pollination drops and are most likely part of the protein degradome that forms as nucellar cells break down. Ubiquitin is one such protein. It has a number of well-established intracellular roles as part of the ubiquitin–proteasome system in plants (Sadanandom et al. 2012). Ubiquitins are involved in the regulation of many intracellular processes including development, cell division, response to hormonal signals and abiotic or biotic stressors (Sadanandom et al. 2012). However, extracellular, i.e. apoplastic, roles for ubiquitin have also been recently shown in plants. Kim et al. (2006) concluded that free ubiquitin found at the tips of lily pollen tubes raised in vitro effectively enhanced the uptake of stigma/stylar cysteine-rich adhesion protein and may therefore affect pollen tube adhesion.

Overall, *Ginkgo biloba* has similar pollination drop proteins to other gymnosperms: carbohydrate-modifying enzymes, proteases, lipid transfer proteins and thaumatin-like proteins. In addition, *G. biloba* produces amino acids and sugars in its pollination drops in a profile that is characteristic of extant insect-pollinated or ambophilous gymnosperms (Nepi et al. 2017). However, *Ginkgo biloba* also holds an important place in our understanding of seed plant evolution (del Tredici 2007). *Ginkgo biloba* is the only living representative of the once diverse order Ginkgoales (Christianson and Jernstedt 2009), which share a number of reproductive traits with an equally ancient group, cycads, such as haustorial pollen tubes and zooidogamy (Chamberlain 1935; Friedman 1987). Although it is perhaps not surprising that the biochemistry of their pollination drops is similar, this does imply that ancient ginkgos and cycads likely had pollination drops and that these drops may have contained a secretome made up of defence proteins and carbohydrate-modifying enzymes.

### Gnetales

Both *Gnetum gnemon* and *Welwitschia mirabilis* are dioecious, with ovulate cones occurring on separate plants from pollen cones as in *Ginkgo* and cycads. In these gnetalean genera, ovulate cones bear fertile ovules that produce pollination drops, and interestingly, pollen cones bear sterile ovules that also produce pollination drops. Our analysis detected proteins in the pollination drops of both *G. gnemon* and *W. mirabilis*, including proteins in the pollination drops of fertile ovules, referred to from now on as female drops, and sterile ovules from pollen cones, henceforth referred to as male drops. For the first time, we can conclude that all three extant gnetalean genera (*Gnetum*, *Welwitschia* and *Ephedra*) have pollination drop proteomes. This is also the first report of proteins occurring in pollination drops collected from the sterile ovules found on pollen cones of any gymnosperm.

#### *Welwitschia*

Five protein hits were recorded in the female drops of *W. mirabilis*, but only one protein was identified, a HOPZ-activated resistance 1 protein with a GO annotation to the nucleus. This protein is involved in recognition of the type III secreted effector protein HopZ1a from the bacteria *Pseudomonas syringae* (Lewis et al. 2010). We did not detect the chitinase protein that Wagner et al. (2007) reported in their analysis of *W. mirabilis*, nor the acid phosphatase found by Carafa et al. (1992). The lack of proteins detected in our study and previous studies may indicate that *W. mirabilis* female drops lack a strong secretome and do not have a strong compliment of antimicrobial proteins. In support of this, in their natural habitat *W. mirabilis* ovules are commonly infected by the fungus *Aspergillus niger* which gains access through pollination drops (Whitaker et al. 2008).

In contrast to the lack of proteins in the female drops, we identified 138 proteins in the male drops of *W. mirabilis.* Most proteins were annotated to intracellular locations (> 90%) and the plasma membrane (> 35%). Annotations to the extracellular space were present in around 35% of the identified proteins; however, *W. mirabilis* male pollination drops lacked the types of proteins that have been attributed to the secretome of other groups. For example, male drops lacked chitinase, galactosidase, glycosyl hydrolase, beta-glucosidase or aspartyl protease that were seen in the female drops of most gymnosperm groups. The proteome of *W. mirabilis* male drops was instead filled by such proteins as are involved in intracellular processes, e.g. translation, protein folding, glycolysis, cytoplasmic protein transport. Male *W. mirabilis* drops appear to be dominated by proteins attributable to a protein degradome.

#### *Gnetum*

Seventeen proteins were identified in *G. gnemon* female drops, most of which were annotated to the extracellular space (> 85%), likely composing a protein secretome. Only six proteins were annotated to the intracellular space. During drop production, a shallow pollen chamber is formed by cells dying in the nucellus (van der Pijl 1953). As in other gymnosperms that form pollen chambers, the release of this cellular debris results in the protein degradome that we detected. The number of proteins identified in *G. gnemon* female drops was similar to those reported for fellow gnetalean *Ephedra* spp. (von Aderkas et al. 2014) which had between six and 20 proteins depending on species.

The secretome proteins of female *G. gnemon* drops were similar to those found in the other gymnosperm groups (O’Leary et al. 2007; Pirone-Davies et al. 2016; Poulis et al. 2005; von Aderkas et al. 2014; Wagner et al. 2007), including a complement of carbohydrate-modifying enzymes such as glucanase, chitinase, galactosidase and glycosyl hydrolase. *G. gnemon* contained an additional enzyme, a fucosidase, which may also be involved in modifying carbohydrate components of the cell wall (Günl et al. 2011). Proteins involved in oxidation–reduction processes were found, for example peroxidase and FAD-binding Berberine family protein. Osmotin and aspartyl protease proteins were all identified in female *G. gnemon* drops, each of which has been identified in at least one other gymnosperm group.

An extracellular cystatin was also detected in *G. gnemon* female drops. Plant cystatins are protease inhibitors involved in defence against pathogens (Popovic et al. 2013). Cystatins may also control degradation activity of plant proteases (Szewinska et al. 2013) and regulate programmed cell death through control of associated proteases (Solomon et al. 1999).

We identified about two dozen proteins in the drops collected from the male cones of *G. gnemon*, which was slightly more than the number found in the female drops. However, this was far fewer than the more than eleven dozen proteins identified in male drops of *W. mirabilis*. Similar to *W. mirabilis* male drops, the majority of proteins in *G. gnemon* male drops had intracellular annotations (> 80%) and annotations to the plasma membrane (~ 20%). Only seven proteins (~ 28%) had annotations to the extracellular space.

Annotations for cellular process were highly varied for the proteins in *G. gnemon* male drops. A number of proteins involved in intracellular processes were identified: ATPase subunit 1, ER-type Ca^2+^-pumping ATPase, GTP-binding elongation factor Tu family protein, amongst others. Like the male drops of *W. mirabilis*, it seems that *G. gnemon* male drops also have a strong degradome component. However, some proteins in the male drop *G. gnemon* sample are similar to those attributed to the protein secretome found in *G. gnemon* female drops. FAD-binding Berberine family protein, serine carboxypeptidase-like 35 protein, and cysteine proteinases superfamily protein were generally in common, a result that supports the view that secretomes are fairly conservative amongst gymnosperms.

#### Similarities and differences between gnetalean male and female drops

The higher proportions of intracellular and plasma membrane proteins found in male drops compared to female drops of *W. mirabilis*, and *G. gnemon* may be a further reflection of a functional difference between male and female drops. Both *W. mirabilis* and *G. gnemon* are insect-pollinated: the sugar-rich drops are the reward in this system, and therefore, in terms of ecosystems services, the pollination drops are unequivocally nectar (Koptur 1992). There are many thousands of species of flowering plants that produce nectar (Marazzi et al. 2013), including those that have male and female floral nectar. Male and female nectaries differ in volume of nectar that each secretes (Ashworth and Galetto 2002) and in the physiological dynamics of nectar production (Nepi et al. 2001, 2012), as well as in their proteomes (Chatt et al. 2018). In gymnosperms, we observe similar sex-related differences in nectar. In *Gnetum* and *Welwitschia*, male drops had similar relative percentages of major sugars as female drops, but males had an overall lower total sugar content (Nepi et al. 2017). Consequently, pollinators are provided with the same profile of sugars in both male and female drops, but male drops are ‘cheaper’. Not only are they smaller, they are also less sugar rich, which results in a lower viscosity; they tend to drip from the male ovules and run down onto the collars of the strobili (Gong et al. 2016). There are also differences in the behaviour of male and female drop nectar. In *W. mirabilis*, male drops are not retracted, whereas female drops are secreted and retracted each day (Wetschnig and Depisch 1999). The manner of production of pollination drops may differ in sterile ovules compared with fertile ovules. For example, we do not know whether the extensive degradome in sterile ovules of *W. mirabilis* is due to the formation of a pollen chamber. Along with a failure to initiate sporogenesis, what other functions have been modified or lost in the nucellus of a sterile ovule? In this respect, a future comparative immunohistochemical study of pollination drop production in fertile and sterile ovules could provide interesting answers.

### Protein identification

The discussion has centred on proteins that have been annotated based on BLASTp hits that meet an *E*-value threshold. However, it is worth noting in this proteomic survey that numerous proteins were detected that had poor BLASTp matches, or no matching gene at all. Such unknowns are common in proteomic surveys, particularly in the case of non-model species, such as any gymnosperm species. The number of peptides that did not produce identifications varied by species: *Ceratozamia hildae*—8, *Zamia furfuracea*—3, *Cycas rumphii*—6, *Gnetum gnemon* female drops—7, *G. gnemon* male drops—5, *Welwitschia mirabilis* female drops—5 and *W. mirabilis* female drops—5. There are limitations inherent in the methodology. For example, when high-quality mass spectra data from gymnosperms have been processed through PEAKS, we get reliable and repeatable peptide data. When these data are used for a BLASTp search, the results are often dissatisfying, because of the focus on model organisms amongst available sequences. It is likely that identities for our unidentified proteins will be found as proteomic and genomic databases and their accompanying tools continue to improve and expand in taxonomic breadth.

## Conclusion

Proteins are a common feature of pollination drops in all extant gymnosperm lineages: *Ginkgo biloba*; cycads (*Zamia furfuracea*, *Ceratozamia hildae*, *Cycas rumphii*); Gnetales (*Gnetum gnemon*, *Welwitschia mirabilis*, *Ephedra* spp. (von Aderkas et al. 2014); and conifers (Nepi et al. 2009). In our analyses, the number of proteins detected varied between groups as did the specific proteins identified. However, several types of proteins were shared amongst the groups in our study, and in gymnosperms previously sampled. The ubiquity of carbohydrate-modifying proteins and defence proteins suggests their presence may be a conservative feature amongst gymnosperms (Poulis et al. 2005; Wagner et al. 2007; O’Leary et al. 2007).

Many of the identified pollination drop proteins were annotated to the plant apoplast and are thought to compose a protein secretome. The source of pollination drop proteins is the nucellus, a specialized reproductive tissue that almost certainly has a unique set of expressed genes differentiating it from other tissues involved in secretion to the apoplast, e.g. leaf, root, stigma, style. Most gymnosperm pollen germinates against and grows through the nucellus (except in *Ginkgo* and cycads), but still within the pollination drop or remnants of the drop (Chesnoy 1993), and studies have suggested pollination drops may mediate or control pollen germination and pollen tube growth (von Aderkas et al. 2012; Williams 2012). It is likely that pollination drop proteins may influence these reproductive processes. Therefore, although there is overlap in the types of proteins present in plant apoplastic fluids and pollination drops, the functions of these proteins need to be considered within the context of the nucellar tissue that secretes them.

Our proteomic analyses of pollination drops have shown that all extant gymnosperms share complex reproductive secretions containing proteins. From this, we can infer that the composition of these ancient reproductive secretions was always complex and represents a major component in the pollen capture mechanisms that allowed seed plants to diversify and achieve ecological dominance over geologic time.

### **Author contribution statement**

NAP, SAL, CH, PBT, PvA, PG and DWS helped collect materials. NAP, SAL and IGB analysed data. DWS and CPD supplied databases and ideas. PvA conceived the project. NAP was lead scientist and wrote the paper.

## Electronic supplementary material

Below is the link to the electronic supplementary material.

Supplementary material 1 (DOCX 17 kb)

Supplementary material 2 (DOCX 508 kb)

Supplementary material 3 (XLSX 60 kb)
