# Supplementary material for: Complex reproductive secretions occur in all extant gymnosperm lineages: a proteomic survey of gymnosperm pollination drops
Source: Plant Reprod. 2018 Nov 14;32(2):153–66. doi: 10.1007/s00497-018-0348-z (PMC6500509; doi:10.1007/s00497-018-0348-z)
Supplement: Supplementary file 1 — Supplementary material 1 (DOCX 17 kb) [file 497_2018_348_MOESM1_ESM.docx]

**Title:** Complex reproductive secretions occur in all extant gymnosperm lineages: A proteomic survey of gymnosperm pollination drops

**Journal:** Plant Reproduction

**Authors:** Natalie Prior, Stefan A. Little, Ian Boyes, Patrick Griffith, Chad Husby, Cary Pirone-Davies, Dennis W. Stevenson, P. Barry Tomlinson, Patrick von Aderkas

**Corresponding author:** Patrick von Aderkas, University of Victoria, [pvonader@uvic.ca](mailto:pvonader@uvic.ca)

**Online Resource 1.** Pollination drop sample collection, including species and number of plants

| **Group** | **Species  (no. plants)** | **Collection Date*** | **Collection Location** | **Collectors** |
| --- | --- | --- | --- | --- |
| Cycads | *Ceratozamia hildae* (17) | May 2011 | Montgomery Botanical Center, Coral Gables, FL & Wm Tang’s private collection, Miami, FL | Natalie Prior  Wm Tang  Chad Husby |
|  | *Cycas rumphii* (1)  Accession 92399*A | May 2011 | Montgomery Botanical Center, Coral Gables, FL | Natalie Prior  Chad Husby |
|  | *Zamia furfuracea*  4 plants; accessions  20010213*B  20010213*E  20010214*A  20010214*H | July 2011 | Montgomery Botanical Center | Natalie Prior,  Chad Husby |
| Gingko | *Ginkgo biloba* (4) | April 2012 | University of California at Davis, Davis, CA | Stefan Little |
| Gnetales | *Gnetum gnemon,* fertile ovules (1) | May 2011 | Nolan Glasshouse, New York Botanical Garden, Bronx, New York | Dennis W. Stevenson |
|  | *Gnetum gnemon,* ovules from pollen bearing cones (2) | February 2014 | National Tropical Botanical Garden, Kampong Property, Coconut Grove, Florida | P. Barry Tomlinson  Patrick von Aderkas  Natalie Prior |
|  | *W. mirabilis,* fertile ovules from female bearing cones (1) | May 2011 | Nolan Glasshouse, New York Botanical Garden, Bronx, New York | Dennis W. Stevenson |
|  | *W. mirabilis,* fertile ovules and sterile ovules from pollen bearing cones (2) | May 2011 | Sciences Laboratory Building Greenhouse and the Botanical Conservatory at the University of California at Davis, Davis, California | Stefan Little |

* collections were carried out over the course of the pollination drop production period. In most species, pollination drop volume was low, which necessitated pooling samples to achieve the threshold minimum volume required for mass spectrometric analysis.
