# Supplementary material for: Complex reproductive secretions occur in all extant gymnosperm lineages: a proteomic survey of gymnosperm pollination drops
Source: Plant Reprod. 2018 Nov 14;32(2):153–66. doi: 10.1007/s00497-018-0348-z (PMC6500509; doi:10.1007/s00497-018-0348-z)
Supplement: Supplementary file 2 — Supplementary material 2 (DOCX 508 kb) [file 497_2018_348_MOESM2_ESM.docx]

**Title:** Complex reproductive secretions occur in all extant gymnosperm lineages: A proteomic survey of gymnosperm pollination drops

**Journal:** Plant Reproduction

**Authors:** Natalie Prior, Stefan A. Little, Ian Boyes, Patrick Griffith, Chad Husby, Cary Pirone-Davies, Dennis W. Stevenson, P. Barry Tomlinson, Patrick von Aderkas

**Corresponding author:** Patrick von Aderkas, University of Victoria, [pvonader@uvic.ca](mailto:pvonader@uvic.ca)

**Online Resource 2.** Sources and species of transcriptomic data used to create the Gymno_DB database.

Wickett NJ, Mirarab S, Nguyen N, Warnow T, Carpenter E, Matasci N, Ayyampalayam S, Barker MS, Burleigh JG, Gitzendanne MA, Wong GK, Leebens-Mack J ( 2014). Phylotranscriptomic analysis of the origin and early diversification of land plants. Proc Natl Acad Sci USA 111: e4859-e4868. PMID: 25355905. https://doi.org/10.1073/pnas.1323926111

Matasci N, Hung LH, Yan Z, Carpenter EJ, Wickett NJ, Mirarab S, Nguyen N, Warnow T, Ayyampalayam S, Barker M, Leebens-Mack J, Wong GK. (2014) Data access for the 1,000 Plants (1KP) project. GigaSci 3: 17. PMID: 25625010. https://doi.org/10.1186/2047-217X-3-17

Xie Y, Wu G, Tang J, Luo R, Patterson J, Liu S, Huang W, He G, Gu S, Li S, Wong GK, Wang J,. (2014). SOAPdenovo-Trans: de novo transcriptome assembly with short RNA-Seq reads. Bioinformatics 30:1660-1666. PMID: 24532719. https://doi.org/10.1093/bioinformatics/btu077

Johnson MT, Carpenter EJ, Tian Z, Bruskiewich R, Burris JN, Carrigan CT, Chase MW, Clarke ND, Covshoff S, dePamphilis CW, Wong GK (2012) Evaluating methods for isolating total RNA and predicting the success of sequencing phylogenetically diverse plant transcriptomes. PLoS One 7:e50226. PMID: 23185583. https://doi.org/10.1371/journal.pone.0050226

| **Source: 1KP (downloaded October 16 2013)** | | | | | | | | | |
| --- | --- | --- | --- | --- | --- | --- | --- | --- | --- |
| https://sites.google.com/a/ualberta.ca/onekp/ | | | | |  | | |  | |
| Illumina | | | | | | | | | |
| **Clade** | **Family** | | **Species** | **Tissue Type** | **Sample Provider** | | | **RNA Extractor** | |
| Conifers | Araucariaceae | | *Agathis robusta* | leaf | D. W. Stevenson | | | D.W. Stevenson | |
| Conifers | Araucariaceae | | *Araucaria rulei* |  | J. Leebens-Mack | | | J. Leebens-Mack | |
| Conifers | Araucariaceae | | *Araucaria sp.* | young Leaf | D. W. Stevenson | | | D.W. Stevenson | |
| Conifers | Araucariaceae | | *Wollemia nobilis* | leaves | S. Graham | | | S. Graham | |
| Conifers | Cephalotaxaceae | | *Amentotaxus argotaenia* | leaf | D. W. Stevenson | | | D. W. Stevenson | |
| Conifers | Cephalotaxaceae | | *Cephalotaxus harringtonia* | leaves | S. Graham | | | S. Graham | |
| Conifers | Cephalotaxaceae | | *Cephalotaxus harringtonia* | branch apex including needles | M. Deyholos | | | M. Deyholos | |
| Conifers | Cupressaceae | | *Athrotaxis cupressoides* | leaf | S. Graham | | | L. DeGironimo | |
| Conifers | Cupressaceae | | *Austrocedrus chilensis* | leaves | S. Graham | | | S. Graham | |
| Conifers | Cupressaceae | | *Callitris gracilis* |  | A. Lowe | | | BGI | |
| Conifers | Cupressaceae | | *Callitris macleayana* |  | A. Lowe | | | BGI | |
| Conifers | Cupressaceae | | *Calocedrus decurrens* | leaves | S. Graham | | | S. Graham | |
| Conifers | Cupressaceae | | *Chamaecyparis lawsoniana* | leaf | D. W. Stevenson | | | BGI | |
| Conifers | Cupressaceae | | *Cryptomeria japonica* | branch apex including needles and cones | M. Deyholos | | | M. Deyholos | |
| Conifers | Cupressaceae | | *Cryptomeria japonica* | leaf | D. W. Stevenson | | | BGI | |
| Conifers | Cupressaceae | | *Cunninghamia lanceolata* | branch apex incl. needles | M. Deyholos | | | M. Deyholos | |
| Conifers | Cupressaceae | | *Cunninghamia lanceolata* | young shoot | S. Graham | | | BGI | |
| Conifers | Cupressaceae | | *Cupressus dupreziana* |  | J. Leebens-Mack | | | J. Leebens-Mack | |
| Conifers | Cupressaceae | | *Diselma archeri* | leaf | D. W. Stevenson | | | D. W. Stevenson | |
| Conifers | Cupressaceae | | *Fokienia hodginsii* | leaf | D. W. Stevenson | | | D. W. Stevenson | |
| Conifers | Cupressaceae | | *Glyptostrobus pensilis* | young shoot | S. Graham | | | BGI | |
| Conifers | Cupressaceae | | *Juniperus scopulorum* | young shoot | S. Graham | | | BGI | |
| Conifers | Cupressaceae | | *Metasequoia glyptostroboides* | leaves | S. Graham | | | S. Graham | |
| Conifers | Cupressaceae | | *Microbiota decussata* | leaves | S. Graham | | | S. Graham | |
| Conifers | Cupressaceae | | *Neocallitropsis pancheri* |  | J. Leebens-Mack | | | J. Leebens-Mack | |
| Conifers | Cupressaceae | | *Papuacedrus papuana* | leaf | D. W. Stevenson | | | D. W. Stevenson | |
| Conifers | Cupressaceae | | *Pilgerodendron uviferum* | leaves | S. Graham | | | S. Graham | |
| Conifers | Cupressaceae | | *Platycladus orientalis* | leaves | S. Graham | | | S. Graham | |
| Conifers | Cupressaceae | | *Sequoia sempervirens* | branch apex including needles | M. Deyholos | | | M. Deyholos | |
| Conifers | Cupressaceae | | *Sequoiadendron giganteum Glaucum* | leaf | D. W. Stevenson | | | BGI | |
| Conifers | Cupressaceae | | *Taiwania cryptomerioides* | leaves | S. Graham | | | S. Graham | |
| Conifers | Cupressaceae | | *Taxodium distichum* | leaves | S. Graham | | | S. Graham | |
| Conifers | Cupressaceae | | *Tetraclinis sp.* |  | J. Leebens-Mack | | | J. Leebens-Mack | |
| Conifers | Cupressaceae | | *Thuja plicata* | young shoot | S. Graham | | | BGI | |
| Conifers | Cupressaceae | | *Thujopsis dolabrata* | young shoot | S. Graham | | | BGI | |
| Conifers | Cupressaceae | | *Widdringtonia cedarbergensis* | young leaf | D. W. Stevenson | | | L. DeGironimo | |
| Conifers | Pinaceae | | *Abies lasiocarpa* | leaf | D. W. Stevenson | | | BGI | |
| Conifers | Pinaceae | | *Cathaya argyrophylla* |  | J. Leebens-Mack | | | J. Leebens-Mack | |
| Conifers | Pinaceae | | *Cedrus libani* | young shoot | S. Graham | | | BGI | |
| Conifers | Pinaceae | | *Keteleeria evelyniana* | young shoot | J. Leebens-Mack | | | J. Leebens-Mack | |
| Conifers | Pinaceae | | *Larix speciosa* | young shoot | S. Graham | | | S. Graham | |
| Conifers | Pinaceae | | *Nothotsuga longibracteata* |  | J. Leebens-Mack | | | J. Leebens-Mack | |
| Conifers | Pinaceae | | *Picea engelmannii* | leaf | D. W. Stevenson | | | BGI | |
| Conifers | Pinaceae | | *Pinus jeffreyi* | stems and leaves | N. Stewart | | | N. Stewart | |
| Conifers | Pinaceae | | *Pinus parviflora* | leaf | D. W. Stevenson | | | D. W. Stevenson | |
| Conifers | Pinaceae | | *Pinus ponderosa* | stems and leaves | N. Stewart | | | N. Stewart | |
| Conifers | Pinaceae | | *Pinus radiata* | leaf | D. W. Stevenson | | | D. W. Stevenson | |
| Conifers | Pinaceae | | *Pseudolarix amabilis* |  | J. Leebens-Mack | | | J. Leebens-Mack | |
| Conifers | Pinaceae | | *Pseudotsuga wilsoniana* | young shoot | S. Graham | | | BGI | |
| Conifers | Pinaceae | | *Tsuga heterophylla* | leaf | D. W. Stevenson | | | BGI | |
| Conifers | Podocarpaceae | | *Acmopyle pancheri* |  | J. Leebens-Mack | | | J. Leebens-Mack | |
| Conifers | Podocarpaceae | | *Dacrycarpus compactus* | leaf | D. W. Stevenson | | | D. W. Stevenson | |
| Conifers | Podocarpaceae | | *Dacrydium balansae* |  | J. Leebens-Mack | | | J. Leebens-Mack | |
| Conifers | Podocarpaceae | | *Falcatifolium taxoides* |  | J. Leebens-Mack | | | J. Leebens-Mack | |
| Conifers | Podocarpaceae | | *Falcatifolium taxoides* |  | J. Leebens-Mack | | | J. Leebens-Mack | |
| Conifers | Podocarpaceae | | *Halocarpus bidwillii* | leaves | S. Graham | | | S. Graham | |
| Conifers | Podocarpaceae | | *Lagarostrobos franklinii* | young end shoots | M. Ruhsam | | | M. Ruhsam | |
| Conifers | Podocarpaceae | | *Manoao colensoi* | leaf | D. W. Stevenson | | | BGI | |
| Conifers | Podocarpaceae | | *Microcachrys tetragona* | leaves | S. Graham | | | S. Graham | |
| Conifers | Podocarpaceae | | *Microstrobos fitzgeraldii* | young shoot | D. W. Stevenson | | | BGI | |
| Conifers | Podocarpaceae | | *Nageia nagi* | leaf | D. W. Stevenson | | | BGI | |
| Conifers | Podocarpaceae | | *Parasitaxus usta* |  | J. Leebens-Mack | | | J. Leebens-Mack | |
| Conifers | Podocarpaceae | | *Phyllocladus hypohyllus* | leaf | D. W. Stevenson | | | D. Stevenson | |
| Conifers | Podocarpaceae | | *Podocarpus coriaceus* | leaf | P. Thomas | | | D. W. Stevenson | |
| Conifers | Podocarpaceae | | *Podocarpus rubens* | leaf | P. Thomas | | | D. W. Stevenson | |
| Conifers | Podocarpaceae | | *Prumnopitys andina* | young shoot | S. Graham | | | BGI | |
| Conifers | Podocarpaceae | | *Retrophyllum minus* |  | J. Leebens-Mack | | | J. Leebens-Mack | |
| Conifers | Podocarpaceae | | *Saxegothaea conspicua* | young shoot | D. W. Stevenson | | | BGI | |
| Conifers | Podocarpaceae | | *Sundacarpus amarus* |  | D. Soltis | | | D. Soltis | |
| Conifers | Sciadopityaceae | | *Sciadopitys verticillata* | young shoot | S. Graham | | | BGI | |
| Conifers | Taxaceae | | *Austrotaxus spicata* |  | J. Leebens-Mack | | | J. Leebens-Mack | |
| Conifers | Taxaceae | | *Pseudotaxus chienii* |  | J. Leebens-Mack | | | J. Leebens-Mack | |
| Conifers | Taxaceae | | *Taxus baccata* | mature leaves and small woody branch | M. Deyholos | | | M. Deyholos | |
| Conifers | Taxaceae | | *Taxus cuspidata* | branch apex including needles | M. Deyholos | | | M. Deyholos | |
| Conifers | Taxaceae | | *Torreya nucifera* | young shoot | S. Graham | | | BGI | |
| Conifers | Taxaceae | | *Torreya taxifolia* | young shoots | J. Leebens-Mack | | | J. Leebens-Mack | |
| Cycadales | Cycadaceae | | *Cycas micholitzii* | leaves | Tao Chen | | | BGI | |
| Cycadales | Stangeriaceae | | *Stangeria eriopus* | young Leaf | D. W. Stevenson | | | D.W. Stevenson | |
| Cycadales | Zamiaceae | | *Dioon edule* | leaf | D. W. Stevenson | | | BGI | |
| Cycadales | Zamiaceae | | *Encephalartos barteri* |  | D. W. Stevenson | | | BGI | |
| Ginkgoales | Ginkgoaceae | | *Ginkgo biloba* | developing shoots | D. W. Stevenson | | | M. Deyholos | |
| Gnetales | Ephedraceae | | *Ephedra sinica* | shoot | M. Deyholos | | | M. Deyholos | |
| Gnetales | Gnetaceae | | *Gnetum montanum* | leaves | Tao Chen | | | BGI | |
| Gnetales | Welwitschiaceae | | *Welwitschia mirabilis* |  | J. Leebens-Mack | | | J. Leebens-Mack | |
|  |  | |  |  |  | | |  | |
| **Source: unpublished data from the New York Plant Genomics Consortium. United States National Science Foundation grant: IOS-0922738 (Downloaded April 2013)** | | | | | | | | | |
| Illumina |  |  | |  |  | | |  | |
| **Clade** | **Family** | **Species** | | **Tissue Type** |  | | |  | |
| Conifers | Cupressaceae | *Metasequoia* | | young ovules, seeds, young leaves |  | | |  | |
| Ginkgoales | Ginkgoaceae | *Ginkgo biloba* | | young ovules, seeds, young leaves |  | | |  | |
| Cycadales | Cycadaceae | *Cycas rumphii* | | leaves |  | | |  | |
|  |  |  | |  |  | | |  | |
| **Source: Dendrome (downloaded April 30 2013)** | | | |  |  | | |  | |
| 454 Assemblies | |  | |  |  | | |  | |
| http://dendrome.ucdavis.edu/treegenes/transcriptome/transcr_summary.php | | | | | | |  |  | |
| http://loblolly.ucdavis.edu/bipod/ftp/Transcriptome_Data/454/ | | | | |  | | |  | |
| **Clade** | **Family** | **Species** | |  |  | | |  | |
| Gnetales | Gnetaceae | *Gnetum gnemon* | |  |  | | |  | |
| Conifers | Pinaceae | *Picea abies* | |  |  | | |  | |
| Conifers | Pinaceae | *Pinus lambertiana* | |  |  | | |  | |
| Conifers | Pinaceae | *Pinus taeda* | |  |  | | |  | |
| Conifers | Podocarpaceae | *Podocarpus macrophyllus* | |  |  | | |  | |
| Conifers | Pinaceae | *Pseudotsuga menziesii* | |  |  | | |  | |
| Conifers | Sciadopityaceae | *Sciadopitys verticillata* | |  |  | | |  | |
| Conifers | Cupressaceae | *Sequoia sempervirens* | |  |  | | |  | |
| Conifers | Taxaceae | *Taxus baccata* | |  |  | | |  | |
|  |  |  | |  |  | | |  | |
| **Source: Data from the Natural Sciences and Engineering Research Council of Canada Strategic Grant *Megastigmus* and Conifers: The Biology of Invasion (downloaded April 2013):**  Little SA, Boyes IG, Donaleshen K, von Aderkas P, Ehlting J (2016) A transcriptomic resource for Douglas-fir seed development and analysis of transcription during late megagametophyte development. Plant Reprod 29:273-286 | | | | | | | | | |
| **Clade** | **Family** | **Species** | | **Tissue Type** | | **Sample Provider** | | | **RNA Extractor** |
| Conifers | Pinaceae | *Pseudotsuga menziesii* | | Megagametophyte, nucellus, cone scale complexes | | Ian Boyes | | | Ian Boyes |
|  |  |  | |  | |  | | |  |
